# No evidence for confounding orientation-dependent fixational eye movements under baseline conditions

**DOI:** 10.1038/s41598-018-30221-2

**Published:** 2018-08-03

**Authors:** Jordy Thielen, Rob van Lier, Marcel van Gerven

**Affiliations:** Radboud University, Donders Institute for Brain, Cognition and Behaviour, Nijmegen, The Netherlands

## Abstract

Decoding has become a standard analysis technique for contemporary cognitive neuroscience. Already more than a decade ago, it was shown that orientation information could be decoded from functional magnetic resonance imaging voxel time series. However, the underlying neural mechanism driving the decodable information is still under debate. Here, we investigated whether eye movements and pupil dilation during attempted fixation and passive viewing of visually presented square-wave grating stimuli could explain orientation decoding. We hypothesized that there are confounding orientation-dependent fixational eye movements (e.g., microsaccades), which systematically alter brain activity, and hence can be the source of decodable information. We repeated one of the original orientation decoding studies, but recorded eye movements instead of brain activity. We found no evidence that stimulus orientation can be decoded from eye movements under baseline conditions, but cannot rule out the potential confounding effect of eye movements under different conditions. With this study, we emphasize the importance, and show the implications of such potential confounding eye movements for decoding studies and cognitive neuroscience in general.

## Introduction

Nowadays, multivariate pattern analysis (MVPA) is one of the standard methods to study brain function^1^. Such decoding model can learn complex transformations to recover perceptual or cognitive states from recorded brain activity. Examples are decoding of stimulus orientation^2,3^, object category^4^, or perceived characters^5^. Decoding strategies are sensitive to weak, distributed patterns of brain activity that can remain unnoticed in univariate methods. However, the interpretation of decoding results remains controversial and susceptible to misinterpretations^6–9^.

A notorious example of such controversy is the longstanding debate about the source of orientation decoding. One of the first applications of MVPA involved the decoding of perceived stimulus orientation from patterns of blood oxygenation level dependent (BOLD) activation recorded with functional magnetic resonance imaging (fMRI). Accurate orientation decoding has been shown for visually presented grating stimuli^2,3^, and also for those held in visual working memory^10^. These results might seem obvious, because it has long been known that primary visual cortex is organized in orientation columns, where individual neurons are tuned to specific orientations^11^. However, it has also been shown that these orientation columns are represented at a much finer spatial scale in the brain, than the typical voxel resolution in fMRI^12^. The population of neurons contained within one voxel could therefore represent the full range of possible orientations uniformly. This renders it unlikely that orientation tuning is a source of decodable information in these orientation decoding studies.

To date, several theories have been proposed to account for why it might be possible to decode orientations from BOLD activation. Firstly, the fine scale bias account suggests a sampling bias of orientation selective neurons within individual voxels^2,3,13^. Here, it is postulated that weak biases emerge because of the imperfect alignment of voxels with the columnar organization of visual cortex, which could give rise to an orientation preference at the voxel level and thus support orientation decoding. These small biases might be too weak to be picked up by univariate analyses, but MVPA can combine many voxels and thereby create a reliable classifier. Secondly, the coarse scale account suggests it is not a local bias at the level of voxels, but instead a large scale bias in the distribution of orientation selective neurons across the entire retinotopic map^14–16^. For instance, a radial bias has been shown, where voxels show a higher response amplitude at peripheral locations that represent orientations toward the fixation point^15,17^. However, other studies have demonstrated robust decoding in the absence of a radial bias, which suggests that a radial bias is not necessary for decoding^18,19^. Thirdly, it has been shown that small biases to orientations arise at the edges of the stimulus, which might drive decodability^20^. This was shown with a model that assumed no biases at the local nor at the coarse scale. While a study showed that this edge related activity is not necessary to explain decoding of orientation^21^, a recent study showed stimulus vignetting, the source for these edge effects, might be the cause of the radial bias, and in turn orientation decoding^22^. It is worth noting that the aforementioned theories each might have a share in the source of decodability, but that after more than a decade this discussion has not yet been resolved and a clear picture is still awaiting.

We propose eye movements as a source of decodable information in orientation decoding. Eye movements cause the retinal image to shift, which in turn alters early visual cortex activations. The effect on visual cortex has been shown to be similar for voluntary eye movements, involuntary eye movements, and artificial image shifts^23^. Additionally, it has been shown that downstream visual areas remain eye movement invariant, representing stable representations of the visual world regardless of eye movements. Instead, early visual areas are strongly affected by eye movements^24^. Thus, when eye movements are systematic, activity in early visual cortex will be systematically altered as well. A decoder could easily pick up these systematic patterns of brain activity. For instance, when the direction of eye movements is tightly coupled to which stimulus is presented, brain activity is evoked that is specific to the presented stimulus, which in turn can be used by a decoder to classify the stimulus. It is important to note that the source of decodable information in this case would have nothing to do with a sensitivity to the presented stimulus (e.g., orientation selectivity), but instead can be completely explained by systematic eye movements.

In most decoding studies, participants are asked to fixate their eyes on a fixation dot to prevent any potential confounding eye movements during the experiment. However, participants’ eyes are never as fixated as one might expect. During attempted fixation, the participants make so-called fixational eye movements. These are small amplitude eye movements around the point of fixation, preventing neuronal adaptation and hence perceptual fading^25–27^. Thus, having these fixational eye movements is a good thing, because otherwise participants would not perceive anything at all. Fixational eye movements can be divided in three classes: microsaccades, drifts, and tremors^27,28^. Microsaccades happen approximately one to two times per second, with a maximum amplitude of 30-minutes of arc, and are correlated between the eyes. Tremor is a 30 to 100 Hz oscillation superimposed on drift, without correlation between eyes. Drift has an amplitude of 30-minutes of arc per second and is not correlated between the eyes. Microsaccades might bring back the eyes to fixation and realign the eyes after drift^26^ and blinks^29^. Binocular microsaccades are predominantly horizontal, and monocular microsaccades are equally expressed in horizontal as well as vertical directions^26^.

Fixational eye movements have been shown to be associated with and thus predictable for certain task-related behaviors. For instance, the direction of microsaccades was shown to be predictive of attentional allocation to either of the hemifields^30^. Interestingly, microsaccades occurred less when attentional load was high, but were then most predictive of the direction of attention, compared to situations of low attentional load where more but less predictive microsaccades occurred. Additionally, microsaccades are predictive of the perceived direction in apparent motion when participants are presented with ambiguous motion patterns^31^. First, there was a drop in the frequency of microsaccades (i.e., microsaccadic inhibition) prior to the perceptual flip of the direction of motion. Second, the direction of microsaccades prior to the presentation of the motion patterns biases the direction of perceived motion.

If orientation-dependent eye movements do exist, this has major implications to previously reported decoding findings. Such confounding eye movements would suggest a completely different explanation of orientation decoding, implying it would have nothing to do with biases across visual cortex at either local or coarse scale. Most importantly, it would signify the potential confounding role of eye movements in decoding studies and cognitive neuroscience in general. Therefore, we emphasize that eye movements should always be considered as a confound in experimental research. To our knowledge, no extensive research has been conducted to investigate whether eye movements could indeed explain several reported (decoding) findings.

In this study, we replicated the procedures from Kamitani and Tong, which is one of the pioneering studies that showed orientation decoding from BOLD activation^3^. Instead of recording brain activity, we recorded binocular eye movements and pupil dilation. We investigated whether it is possible to decode stimulus orientation from these eye movements and pupil dilation. We hypothesized that there are microsaccades orthogonal to the orientation of the presented grating, because of perceived apparent motion due to the random phase that is used for individual presentations of the same oriented grating.

## Methods

### Participants

Fifteen university students (aged 19–30; 13 females) from Radboud University participated in the experiment. Inclusion criteria to participate in this study were age (18–30), handedness (right), and vision capabilities (uncorrected, normal). Exclusion criteria were any history with epilepsy or claustrophobia. All participants gave written informed consent prior to the experiment and received payment or course credit after the experiment. The experimental procedure and methods were approved by and performed in accordance with the guidelines of the local ethical committee of the Faculty of Social Sciences.

### Materials

**Figure 1:**
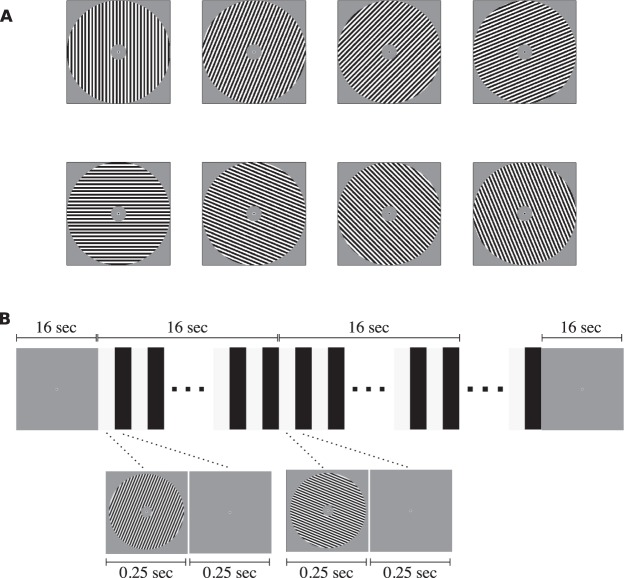
The experimental procedure. (**A**) In the experiment, we presented square-wave gratings with one of eight possible orientations. (**B**) During a trial, one grating was presented at a rate of 2 Hz (i.e., 250 ms on and 250 ms off) for a total duration of 16 seconds. During a trial, the orientation remained the same, but the phase was randomized. A run started and ended with 16 seconds of fixation. In between these fixation periods, eight trials were completed, one for each orientation in random order. Note, the stimuli may be inaccurate because of image quality.

The stimuli were square-wave grating images generated with the exact same parameters as used by Kamitani and Tong^3^. In short, there were eight orientations ranging from angles of 0 to 180 degrees in uniform steps of 22.5 degrees (see Fig. 1). These gratings were full contrast (100 percent) black-white square-wave grating stimuli presented at the center of the screen on a mean-luminance gray background. The outer radius of the grating stimuli subtended 10.0 degree of visual angle (DVA) and the inner radius subtended 1.5 DVA. The spatial frequency was fixed at 1.5 cycles per DVA. The spatial phase was randomized over any presentation of the grating stimuli to ensure that individual pixel information was not informative of any orientation. During the entire experiment, a fixation dot was presented at the center of the screen with an outer radius of 0.15 DVA and an inner radius of 0.075 DVA.

The experiment was run on a Windows desktop PC and the stimuli were presented on a 24 inch BenQ XL2420Z monitor with a 120 Hz frame rate and 1920 × 1080 pixel resolution. The monitor subtended 39.1 DVA horizontally and 22.0 DVA vertically. The experiment was designed and presented with MATLAB (R2015b, The MathWorks, Inc.) using the Psychophysics Toolbox version 3.0.12^32^. An EyeLink 1000 Plus (SR Research, Ltd.) desktop mount eye-tracker was used to record eye positions and pupil dilation. This video-based eye-tracker recorded binocular data with a 35 mm lens at a sample rate of 1000 Hz. The eye-tracker was placed just in front and below the monitor at a distance of 60 cm from the participants’ eyes. Participants’ head position and viewing distance were fixed at 70 cm from the monitor with a chin and forehead rest.

### Procedure

The experimental paradigm was equal to that of Kamitani and Tong^3^ (see Fig. 1). Once written informed consent was given and instructions were clear to the participant, the experiment was started. The task of the participant was to fixate at the center of the screen where the fixation dot was presented, and to passively view the grating stimuli. Participants were informed that individual runs were short and that they could take breaks in between. Participants were not explicitly told to fixate as steady as possible, because microsaccades can be voluntarily suppressed^33^, and to maintain a fair comparison to the procedure by Kamitani and Tong^3^. Participants were kindly asked to keep eye blinks to a minimum to preserve the quality of the eye-tracking data.

The experimental paradigm deviated from Kamitani and Tong^3^ in that the experiment was split over two sessions to limit the duration of individual sessions. This was done to prevent discomfort of the participant because of the fixated posture throughout the experiment. In both sessions, twelve similar runs were presented. In each run, eight trials were presented being each of eight possible orientations once. The order of these trials was randomized for each run.

A run always started and ended with sixteen seconds of fixation, during which only the fixation dot was presented on the screen. In between, the eight trials were presented sequentially, without any inter-trial interval. During each trial, one of eight grating stimuli was flashed at 2 Hz (on/off for 250 ms) for 16 seconds. For each of the on phases, the spatial phase of the presented grating image was randomized. During the off periods, only the fixation dot was presented.

In summary, a run contained eight trials of 16 seconds plus two fixation intervals of 16 seconds at the start and end of the run, summing up to 160 seconds per run. Participants completed 12 of such runs in one session. In between runs, participants could take a break and relax their neck and shoulders. Therefore, the eye-tracker was (re-)calibrated prior to each run. Participants completed two sessions, so twenty-four trials were collected for each orientation. Hence, the number of recorded examples for each orientation is equal to that in Kamitani and Tong^3^.

### Preprocessing

The eye-tracking data were recorded at a sample rate of 1000 Hz and saved for offline analysis. The data consisted of binocular (*x*, *y*) positions and pupil dilation. Missing values caused by eye blinks were replaced by medians (i.e., the fixation point). Missing data occurred on average in 2 percent of the data, and were not correlated with the presented orientation. The data were then low-pass filtered using a Butterworth filter of order 5 and a cutoff frequency at 100 Hz. The data were then downsampled from the original 1000 Hz to 256 Hz. Finally, the data were sliced to individual 16 second trials synchronized to the onset of the first stimulus presentation in a trial. Before subjecting the data to the analyses, medians were subtracted for individual runs, to center the data around the fixation point and remove any bias caused by recalibration at the start of runs.

### Analysis

Five analyses were carried out to study any systematics in the recorded eye movement data. Analyses were written in Python (version 2.7.13) using the scikit-learn module (version 0.18.1), a module for machine learning, and the chainer module (version 3.2.0), a module for artificial neural networks. For any of these analyses, the aim was to decode the orientation of the presented grating from the binocular eye movement and pupil dilation time series. These five analyses included (1) decoding with a support vector machine (SVM), (2) decoding summary statistics with a SVM, (3) decoding individual time points with a SVM, (4) decoding with a convolutional neural network (CNN), and (5) decoding with a recurrent neural network (RNN). The following sections describe the analyses in more detail.

For each of these analyses, data included both eyes’ (*x*, *y*) position and pupil dilation during the 16 second trials. A training and testing split of the data was made for performance evaluation of the five analyses by means of a leave-one-run-out cross-validation. Hence, a cross-validation was performed using the data of 23 runs for training, and the calibrated classifier was subsequently tested on the left-out run. Data were standardized using the mean and standard deviation from the training split. Chance level performance was at 12.5 percent correct classifications, because there were eight classes (i.e., the orientations of the gratings). Statistical significance at the group level was determined by performing a one-sample one-sided Wilcoxon signed-rank test on difference scores with chance level. Scores of zero (i.e., at chance level) were included in the ranking.

Participant 5 did not complete the full experimental design because of trouble with the calibration of the eye-tracker. This participant’s data was still included in the analyses. For this participant not 24, but 20 runs were recorded.

#### Decoding with a support vector machine

A straightforward approach to decode the orientations from the binocularly recorded eye locations and pupil dilation, would be to use each individual recorded sample as a feature for a one-versus-one linear SVM. In this analysis, we therefore stacked and stored all recorded single-trial time points in a single vector. Thus, the data was of shape $X\in {{\mathbb{R}}}^{k,m}$, with *k* = 192 is the number of trials, and *m* = 2 * 3 * 16 * 256 = 23576 is the number of features (i.e., eye times features times seconds times sample rate).

Unfortunately, such SVM learns one weight for each individual feature at each individual time point to perform classification. This approach might be too simplistic because it ignores the dependency of samples within features. Additionally, and more importantly, eye movements and specifically microsaccades might occur at different points in time, which might be hard to capture in this way. For this reason, we employed several more analyses.

#### Decoding summary statistics with a support vector machine

A common way to analyze confounding effects of eye movements, is to compute summary statistics of the eye movement time series, and use these as input to a one-versus-one linear SVM. Commonly used summary statistics are the mean and standard deviation of the (*x*, *y*) position and pupil dilation time series. However, these might not be sufficient to pick up on systematic eye movements. First, the effects of fixational eye movements might be too small and too infrequent to be picked up by means and standard deviations. Second, we hypothesized that there are systematic eye movements orthogonal to the stimulus orientation. These eye movements might form a straight line centered around the fixation point. A mean would not pick up these systematic characteristics.

Therefore, we extended the summary statistics to not only include the means and standard deviations, but also the angle of the eye relative to fixation and the direction of detected microsaccades. The angle of the eye relative to fixation was expressed as the percentage of time the eye was at a certain angle from the fixation point within a trial. These angles were discretized into 16 bins of 0 to 360 degrees in steps of 22.5 degrees. Microsaccades were detected with the microsaccade detection algorithm of Engbert^34^. In brief, the (*x*, *y*) positions of the eyes were converted to velocities using a moving average derivative of 6 samples. Subsequently, thresholds *σ*_*x*_ and *σ*_*y*_ were defined and used to detect microsaccades when the velocities exceeded these thresholds. Finally, the duration of detected microsaccades had to exceed 6 ms. We detected microsaccades and measured their direction discretized to 16 angles ranging from 0 to 360 degrees in steps of 22.5 degrees.

In total, for this analysis, the data was of shape $X\in {{\mathbb{R}}}^{k,m}$, with *k* = 192 trials, and *m* = 2*(3 + 3 + 16 + 16) = 76 features (i.e., eye times means plus standard deviations plus eye angle plus microsaccade direction).

#### Decoding individual time points with a support vector machine

Another common practice is to analyze time series sample by sample. Here, for each individual time point, both eyes’ (*x*, *y*) position and pupil dilation were directly classified by a one-versus-one linear SVM, thus the data was of shape $X\in {{\mathbb{R}}}^{k,m}$ with *k* = 192 and *m* = 6. The output of this analysis was the classification accuracy over time.

Note, that also this approach might be too simplistic to find effects of fixational eye movements. Fixational eye movements and especially microsaccades are infrequent events that most likely happen at irregular points in time. Interpreting individual time points does assume that fixational eye movements happen at the same time.

#### Decoding with a convolutional neural network

One way to overcome the above mentioned issues is to classify the time series at once. Specifically, a method is required that interprets the time points as individual features and integrates these to make a classification.

A CNN learns a stack of increasingly complex local feature detectors. In its convolutional layers, a CNN learns filters that look for small local patterns in the input space. Specifically, each artificial neuron in a convolutional layer represents a filter that is replicated over spatial locations (i.e., convolution). Hence, such small filter requires only few parameters to be learned, while still the full image can be processed.

Typically, CNNs are used for image recognition (see e.g.^35^), where the input is a 2D image. Here, we used 1D convolution to analyze the eye movement time series. The input to the CNN were the time series of each of 6 features. The CNN had five layers of artificial neurons, of which the first three were convolutional and the last two were fully connected layers. The first convolutional layer had 6 kernels with a size of 32 samples (i.e., 125 ms). The second convolutional layer had 16 filters with a size of 16 samples (i.e., 62.5 ms). The third convolutional layer had 120 kernels of size 8 (i.e., 31.25 ms). The fourth and fifth layers were fully connected layers, first projecting the output of the convolutional layers to a vector of 84 units, and subsequently to 8 output units, one for each orientation. The artificial neurons were rectified linear units (ReLU) which is common for CNNs, only the output layer contained softmax units which is common for classification. After layer one and two, max pooling was performed with a pooling window of size 2 and a stride of 2. Max pooling is typically done to introduce spatial invariance to the input. The CNN was trained in 100 epochs using Adam^36^ with parameters *α* = 0.001, *β*_1_ = 0.9, and *β*_2_ = 0.999.

#### Decoding with a recurrent neural network

Another way to overcome the above mentioned issues is to classify the time series with a recurrent approach. This can be done using a RNN. A RNN does not only process the input of the current time point, but receives an accumulation of previous time points as input too. In this way, a RNN can use both previous time points as well as current input for classification.

Typically, RNNs are used for sequence learning like natural language, predicting the next words in a sequence. Here, we used 20 long short term memory (LSTM^37^) units and a final fully connected layer of eight output units with a softmax activation function. At a time point, the RNN received the six features and the representation of the previous time point as input. The RNN was trained in 100 epochs using Adam^36^ with parameters *α* = 0.001, *β*_1_ = 0.9, and *β*_2_ = 0.999.

### Data availability

Data are available from the Donders Institute for Brain, Cognition and Behaviour repository at http://hdl.handle.net/11633/di.dcc.DSC_2016.00311_772.

## Results

### Decoding with a support vector machine

**Figure 2:**
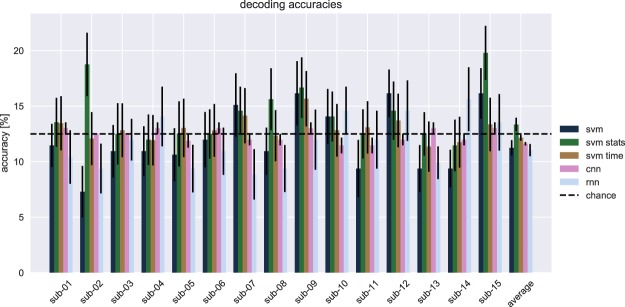
Decoding accuracies. For each of five methods, the decoding accuracies for individual participants and the grand average are shown. Bars represent the average over the leave-one-run-out cross-validation, error bars represent the standard error. Chance level decoding was 12.5 percent correct classifications (dashed line).

The grand average classification accuracies of the SVM directly applied to the eye movements are shown in Fig. 2. The SVM classified the full feature vector with accuracies around chance level (12.5 percent). A permutation test with 1000 permutations and an alpha level of 0.05 was carried out to verify statistically significant decoding accuracy within participants. None of the participants performed higher than chance according to the permutation test ($p > 0.05$, for all participants). Additionally, a one-sided Wilcoxon signed-rank test revealed no greater than chance level performance at the group level (*p* = 0.628).

### Decoding summary statistics with a support vector machine

The grand average classification accuracies of the SVM on summary statistics are shown in Fig. 2. The SVM classified with accuracies around chance level (12.5 percent). For this analysis, we carried out a permutation test with 1000 permutations and an alpha level of 0.05 to verify within participants statistically significant decoding accuracy. This revealed significant decoding accuracies for two participants (sub-02, 18.8 percent, $p < 0.008$; sub-15, 19.8 percent, *p* = 0.004). However, a one-sided Wilcoxon signed-rank test revealed no greater than chance level performance at the group level (*p* = 0.026). A post-hoc analysis was carried out to investigate individual differences. Both age (Pearson’s correlation, *ρ* = 0.22, *p* = 0.167) as well as gender (Mann-Whitney U-test, *p* = 0.171) did not co-vary with the decoding accuracies.

**Figure 3:**
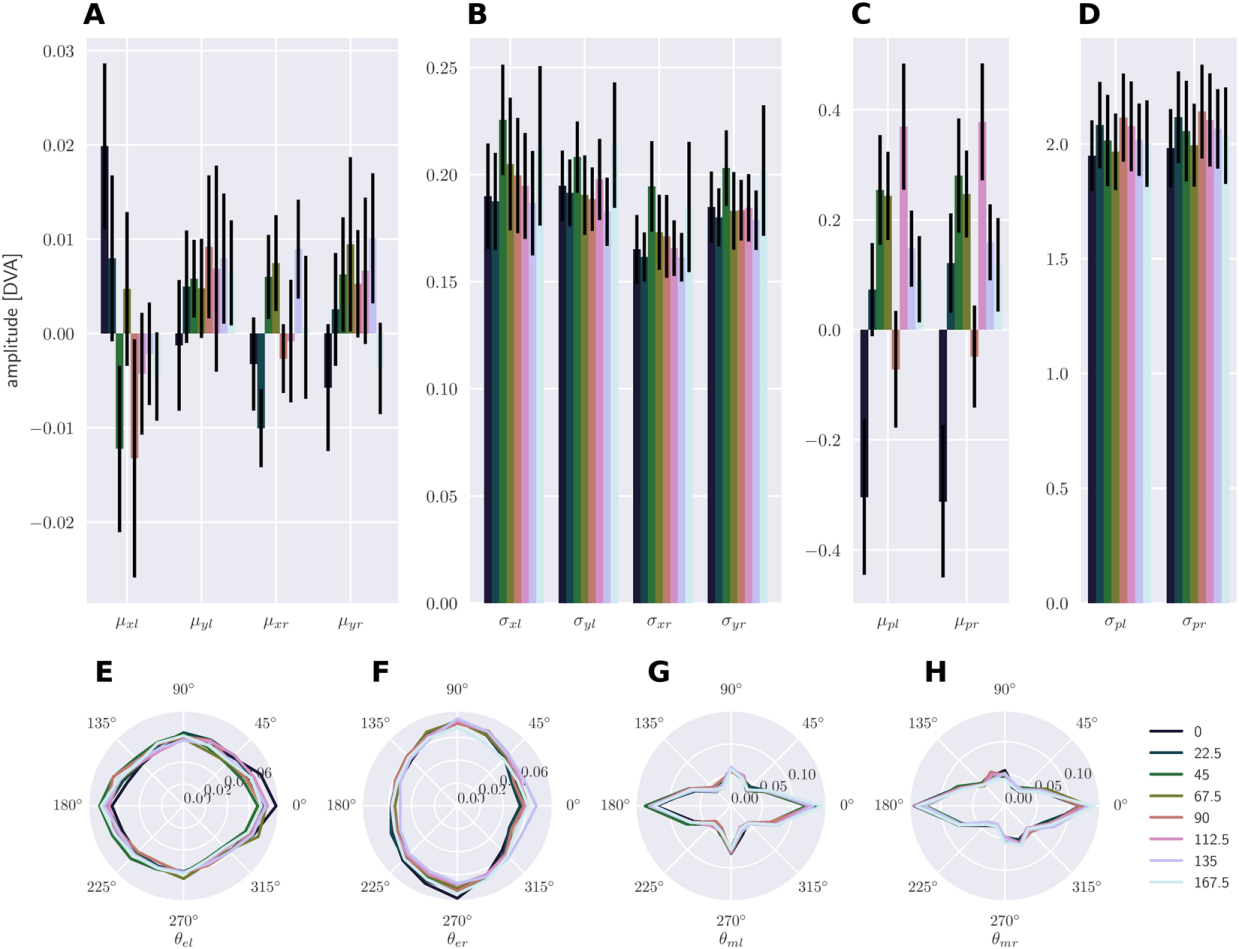
Grand average summary statistics. The summary statistics that were used by the second analysis method (i.e., SVM stats). Bars represent the grand average and error bars represent the standard error over participants. The summary statistics were computed for individual trials and eyes, but here the average summary statistic per orientations is shown. (**A**) *μ*_*xl*_, *μ*_*yl*_, *μ*_*xr*_, and *μ*_*yr*_ are the means, and (**B**) *σ*_*xl*_, *σ*_*yl*_, *σ*_*xr*_, and *σ*_*yr*_ are the standard deviations of the (*x*, *y*) position of both eyes. (**C**) *μ*_*pl*_ and *μ*_*pr*_ are the means, and (**D**) *σ*_*pl*_ and *σ*_*pr*_ are the standard deviations of the pupil dilation of both eyes. (**E**) *θ*_*el*_ and (**F**) *θ*_*er*_ are the distribution of the angle of the eye position relative to the fixation point for both eyes. (**G**) *θ*_*ml*_ and (**H**) *θ*_*mr*_ are the distribution of the direction of microsaccades for both eyes.

Figure 3 shows the grand average and standard errors of the average summary statistics per stimulus orientation. In panel A, *μ*_*xl*_, *μ*_*yl*_, *μ*_*xr*_, and *μ*_*yr*_ are the means, and in panel B *σ*_*xl*_, *σ*_*yl*_, *σ*_*xr*_, and *σ*_*yr*_ are the standard deviations of the (*x*, *y*) position of both eyes. In panel C, *μ*_*pl*_ and *μ*_*pr*_ are the means, and in panel D *σ*_*pl*_ and *σ*_*pr*_ are the standard deviations of the pupil dilation of both eyes. Panels E and F show *θ*_*el*_ and *θ*_*er*_, which are the distributions of the angle of the eye position relative to the fixation point for both eyes. Panels G and H show *θ*_*ml*_ and *θ*_*mr*_, which are the distributions of the direction of microsaccades for both eyes. Both eyes show similar patterns in all subsets of statistics. Differences between orientations’ mean and standard deviation seemed small or non-existent at all for both eyes’ (*x*, *y*) position and pupil dilation. The distribution of the eyes’ angle relative to the fixation point shows a uniform distribution around fixation, and shows no differences between stimulus orientations. The distribution of microsaccade directions shows a bias to horizontal directions, and again no differences between stimulus orientations.

### Decoding individual time points with a support vector machine

We computed the average accuracy over time within participants, and showed these in Fig. 2. This time-average was not statistically greater than chance level according to a one-sided Wilcoxon signed-rank test (*p* = 0.140).

**Figure 4:**
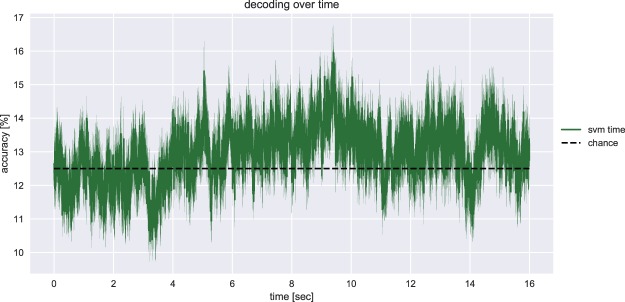
Grand average decoding accuracy over time. The grand average decoding accuracy per time point is shown, which was the output of the third analysis method (i.e., SVM time). Chance level accuracy was 12.5 percent correct classifications (dashed line).

The grand average accuracy over time is shown in Fig. 4. It can be observed that the linear SVM classified individual time points around chance level (12.5 percent). This was confirmed by a Wilcoxon signed-rank test, which revealed no significant decoding accuracies at any time point ($p > 0.025$, Bonferroni corrected, for any time point).

### Decoding with a convolutional neural network

To identify if classification of time series of eye movements benefits a more complex strategy, a CNN was employed. The classification accuracies after training are shown in Fig. 2. It can be observed that the CNN performed around chance level (12.5 percent). This was also confirmed by a one-sided Wilcoxon signed-rank test at the group level that showed no greater than chance level decoding accuracy (*p* = 0.427).

**Figure 5:**
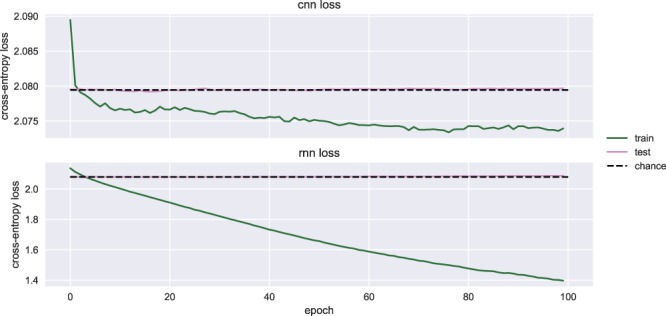
Grand average cross-entropy loss. The grand average over all participants of the cross-entropy loss over training epochs. Both training loss as well as testing loss are shown for both CNN (top panel) and RNN (bottom panel). Chance level cross-entropy loss of a classifier predicting with a uniform probability distribution over classes (i.e., the best one can do if no features are predictive and class labels are balanced) is 2.079 (dashed line).

The cross-entropy loss during the 100 training epochs is shown in Fig. 5. The smaller the cross-entropy loss, the smaller the prediction error the CNN is making, and hence the better the decoding accuracy. The training loss decreased while the validation loss converged to the loss of a random classifier (i.e., the dashed line), the best a classifier could do when there is no predictive information in the data. This is an indication of overfitting, which suggests that the CNN learned patterns specific to the training split (i.e., noise), which did not generalize to the testing split.

### Decoding with a recurrent neural network

Another more complex strategy that was used to classify the eye movement time series was an RNN. The average classification accuracies after training are shown in Fig. 2. It can be observed that the RNN performed around chance level. A one-sided Wilcoxon signed-rank test showed there was no greater than chance level performance at the group level (*p* = 0.210).

The cross-entropy loss during the 100 training epochs is shown in Fig. 5. The training loss still decreased without an improvement in the validation loss, and it can be observed that the validation loss converged to the loss of a random classifier. This suggests the RNN was overfitting on the training data, and learned the characteristics of noise patterns that did not generalize to the testing data.

## Discussion

In this study, we investigated whether the orientation of passively perceived oriented square-wave grating images could be decoded from eye movements and pupil dilation. We replicated the experimental paradigm from Kamitani and Tong^3^, but recorded eye movements instead of brain activity. Participants passively perceived grating stimuli of one of eight possible orientations, whilst fixating on a fixation dot. We analyzed the eye movements in five ways. First, we classified eye movements and pupil dilation directly using a linear SVM. Second, summary statistics of the eye movement time series were classified with a linear SVM. Third, we classified individual time points within the time series with a linear SVM. Fourth, we classified full time series with a CNN. And fifth, we classified full time series with a RNN.

We found no significant decoding accuracies at the group level for any of the analyses. All decoding performances were at chance level and we observed no systematic eye movement parameters (i.e., the summary statistics) under passive viewing. Thus, the data presented here provide no evidence that stimulus orientation can be decoded from eye movements under baseline conditions. Still, based on this null effect, it is a large leap to make the more general claim that eye movements and pupil dilation do not potentially contribute to (orientation) decoding. We will further discuss this in the following sections.

The results of this study do not directly rule out the potential confounding role of eye movements in the study by Kamitani and Tong^3^. We recorded eye movements, while Kamitani and Tong recorded brain activity within an MRI environment. There might be effects of small design changes between the present study and the original study that can mediate eye movements. For instance, the magnetic field, gradient switches, and seated versus lied down position of the participants might have an effect on eye movements. We cannot rule out any effects of such small design changes, although we do believe these are minor. More importantly, the relation between eye movements and BOLD activity is not fully understood, and makes the comparison of decoding results from brain activity to those from eye movements rather difficult. Nonetheless, direct effects of eye movements have been observed in early visual cortex^23,24^. In reality, other predecessors and effects of eye movements (e.g., motor preparation and execution) might inflate the effect of eye movements on brain activity even more.

For two participants, we observed significant decoding accuracies. We suggest that individual differences might mediate these effects. Post-hoc analyses, although limited to age and gender, did not reveal such co-varying variables. Individual differences have previously been shown to affect eye movements. For instance, a suppression of microsaccade rate has been shown around stimulus onset in ADHD patients, which correlated with perceptual variability^38^. Here, we tested a healthy participant population, which is commonly the target population for orientation decoding studies. Note that if individual differences mediate the effect of eye movements, these might become a critical factor in small *N* studies, which is typical for decoding studies.

In this study, a passive viewing paradigm was used. It is possible that when participants do explicitly attend to the stimuli, systematic eye movements might still emerge. Indeed, studies have shown that attention and eye movements are tightly coupled. For instance, microsaccades have been shown to occur more frequently in low attentional load conditions compared to conditions of high attentional load^30,31,39^. Also, it has been shown that these microsaccades are predictive of the attended hemifield^30^, and that the more infrequent microsaccades become, the more predictive they are for perceived direction of ambiguous motion patterns^31^. Additionally, attention has long been known to have effects on pupil dilation too, where tasks with high attentional load or high cognitive demand resulted in larger dilation^40^. Also, it has been shown that covert attention changes the pupillary light response^41^. Pupil dilation has even been shown to reveal an upcoming decision and subjective bias^42^ or value-based decision making in general^43^. Altogether, the involvement of a task that draws attention to the presented stimulus might alter eye movements and pupil dilation in a systematic way, which may create confounding effects.

One study has indeed observed an absence of systematic eye movements in a passive viewing condition, but a presence of confounding eye movements when a task was imposed^44^. In that study, eye movements were recorded during a fMRI study of visual working memory. Systematic eye movements were found when participants were involved in a task requiring them to actively perceive and remember the grating stimuli. The decoding pattern of these eye movements followed a similar pattern as the decoding of the brain activity. This suggests that the decoding pattern might be explained by the eye movements alone, instead of the experimental manipulation. Interestingly, when participants passively viewed the gratings during a functional localizer, no systematic eye movements were observed.

In the current study, the stimuli were replicated from the study as carried out by Kamitani and Tong^3^. Choices in stimulus properties might of course also interact with eye movements. It has been shown that a number of eye movement parameters, like saccade rate, vary with stimulus properties, such as size, under passive viewing^45^. To a broader extent, it might still be possible that systematic eye movements emerge under more natural conditions, such as in the pioneering research on decoding natural object categories^4^.

In all, this study provides no evidence for confounding orientation-dependent eye movements under passive viewing conditions, and hence provides no evidence that orientation decoding might be explained by eye movements. To date, no such extensive study was carried out, regardless of its need to rule out the potential confound. Still, a warning is in place here, as the current study does not completely rule out the potential confounding role of eye movements in any (decoding) study. Individual differences, stimulus properties, task demand, and any experimental manipulation might mediate eye movements. With this study, we want to emphasize and bring under consideration the potential role of confounding eye movements in decoding studies, neuroimaging studies, and in cognitive neuroscience research in general. In every case, researchers should keep an eye on the potential confounding role of eye movements, and for doing so should resort to more extensive analyses.
